# Fairness in AI for healthcare

**DOI:** 10.1016/j.fhj.2024.100177

**Published:** 2024-09-19

**Authors:** Siân Carey, Allan Pang, Marc de Kamps

**Affiliations:** a UKRI CDT for AI in Medical Care and Diagnosis, University of Leeds, UK; b Leeds NHS Teaching Hospitals Foundation Trust, Leeds, UK; c Academic Department of Military Anaesthesia and Critical Care, Royal Centre for Defence Medicine, Birmingham, UK

**Keywords:** Fairness, Artificial intelligence, Machine learning, Bias, Discrimination, Healthcare

## Abstract

Artificial intelligence (AI) is a technology that enables computers to simulate human intelligence and has the potential to improve healthcare in a multitude of ways. However, there are also possibilities that it may continue, or exacerbate, current disparities. We discuss the problem of bias in healthcare and AI, and go on to highlight some of the ongoing and future solutions that are being researched in the area.

## Highlights

• Bias in medical data is prevalent for a wide range of reasons, for example unintended exclusion from randomised clinical trials.
• Bias in artificial intelligence can often arise when it is trained on biased data.
• Educational initiatives will be important for ensuring a wider understanding of AI systems in healthcare.
• It is important that fairness testing and mitigation is built into the process of AI creation.

## Graphical abstract

**Figure:**
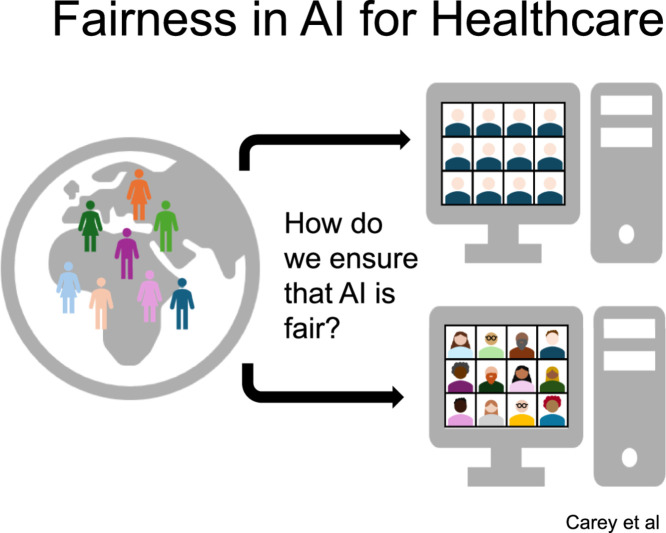

## Introduction

Artificial intelligence (AI) is a technology that enables computers to simulate human intelligence.^1^ With the increasing use of electronic health records, research can be conducted on using AI in a multitude of ways within healthcare. Examples of current work include improving clinical early warning systems,^2^ identification of gold standard treatment plans^3^ and reviews of pathology slides.^4^

Fairness, also known as levels of bias or causes of discrimination, is a frequently defined term.^5^ The common thread of definitions is that an AI model is unfair if there are unjustifiable differences in outcomes due to membership of a marginalised group. A difference in outcomes may be judged by lack of appropriately timed intervention where necessary, as this may affect ongoing quality of life, not available in datasets, rather than just death. A model is fair if it is not unfair. When AI models are unfair, this risks both patient safety and trust from those using and affected by the model. An example of (un)fairness in medical technology is the use of pulse oximeters, which are known to be less accurate on patients with darker skin.^6^

## Background

### Bias in healthcare

AI is a data-driven technology and will reflect the data used to build such models. Unwittingly, medical data contain bias, which, if we are not aware of it, could perpetuate historical bias. The hierarchy of evidence within medical literature rewards the use of randomised controlled trials,^7^ which have inclusion and exclusion criteria to set optimum conditions for measuring treatment effects. The unintended consequence is the tendency to exclude certain subgroups.^8^ Examples include those where informed consent is difficult to obtain (eg patients lacking capacity), where multiple confounding effects are difficult to untangle (eg older population with multi-morbidities) or where it would be unethical to perform experimentation (eg women of childbearing age or children). Exclusion of these groups will result in gaps in knowledge of these particular subgroups.

Even if included in these trials, the reported treatment effects reflect the average treatment effect of the group.^9^ Should there be a difference in effect within a minority subgroup, this will be obscured by the majority group.^10^ This can be detected by post-hoc subgroup analysis; however, being a minority group means that trials are often not powered to detect statistical significance within these groups or require further resources to achieve statistical parity.

The proliferation of digital health records^11^ may offer, via routinely collected data, a partial solution to the selection bias by capturing all population groups receiving healthcare. We should be mindful of the data generation process contained within these systems. While the medical record should be a record of events that could be used for potential future litigation cases, data entry is never truly contemporaneous. It will be retrospective and, therefore, be a filtered/idealised version of events.

Even within data fields that appear objective, such as physiological monitoring, medical equipment can be a source of inaccuracy in different subgroups if not used properly. Examples include inaccurate blood pressure readings with differing body habitus^12^ or inaccurate peripheral oximetry at hypoxic ranges in patients with darker skin.^13^ These inequalities of data quality between groups are inevitable within the medical domain. By understanding these inequalities, we can potentially correct for the bias/uncertainty of models’ outputs.

### Bias in AI

Bias within AI can come from a number of sources, including researcher biases, publishing biases and funding biases. However, a primary source of bias within healthcare AI is data bias.^5^ When discrimination occurs in healthcare settings, it can be visible within patient notes, whether at an individual or mass scale. When these data are given to an AI model, the model is likely to learn and reproduce these biases. Due to the quantity of data required to successfully train and test AI, it is common for data to range back for upwards of 10–20 years. Therefore, even if certain discrimination is not currently occurring, the AI model will still have the opportunity to pick it up from when it was.^14^

If biased AI is introduced into healthcare, there is a risk of increasing the levels of discrimination from what we currently see. This is particularly notable as the outputs of an AI model are likely to be less understandable to an average clinician than the outputs of a current clinical algorithm, so they will not be as able to pick up on these problems themselves.

## Solutions

In this section we will cover potential solutions to the problems raised above. We focus on the problem of unfair AI in healthcare, rather than the broader problem of discrimination in healthcare. This section is split into two parts, considering whether the proposed solution is primarily clinician/organisation led or AI led.

### Healthcare led

Healthcare professionals live by the mantra 'first do no harm’, and so disparities in care are unlikely to be intentional but a result of external factors and unintended consequences in the design of healthcare systems described above. Personal continuous professional development (CPD)^15^ is embedded within the healthcare community and would seem the natural vehicle for delivering educational initiatives to raise awareness of the underlying mechanisms of AI systems. These include the difficulties for models to generalise outside of the training data distribution,^10^ display the level of uncertainty, produce human-understandable explanations for model outputs and, through a fairness lens, the differential performance between different population groups.^16^

We cannot, however, solely put the onus on delivering equitable care on the individual clinician. Ultimately, healthcare is delivered through organisations that are judged by key performance indicators (KPIs) through funding mechanisms such as the Quality and Outcomes Framework (QOF) for primary care and the NHS Payment Scheme (NHSPS) for hospital trusts. QOF attracts funding by providers demonstrating quality metrics of care such as screening rates, clinical outcomes, and best practice process adherence. The NHSPS funding is delivered by levels of activity, such as surgical procedures or courses of treatment. Both systems reward a type of behaviour that attempts to address the clinical need of each domain but can lead to unintended consequences. These include organisations focusing on KPIs with significant funding, diverting attention from other areas of care, or providers 'cherry-picking’ procedures in lower-risk / healthier populations.

This highlights how KPIs could be exploited to address the root causes of inequitable care, but must be done with care to minimise unintended consequences. Developing such KPIs is a challenging balance between having an achievable objective that produces the desired effect of equitable care within the resource-contained environment. Such endeavours require a multidisciplinary approach between front-line clinicians, hospital administrators and computer scientists for these initiatives to be effective and implementable at scale.

### AI led

Both current and potential AI techniques can be implemented to assist in identifying, mitigating and removing bias in AI models. There are a wider range of statistical tests that can be used to identify bias in the results of AI models; these are well established, and commonly focus on one area of fairness concern. For example, treatment equality considers the misclassification rate, and statistical parity considers the difference in outcome.^14^ Therefore, it is important that the pairing of a fairness test to an AI model and task is thoughtfully done to ensure that the concerns are being correctly addressed. These methods are easy to implement and understand; however, they can easily be used inappropriately, and each individual test has its own set of limitations.

Mitigation of bias in AI models can be completed using a number of methods. Data augmentation commonly uses real-world data as a base to then build a less biased dataset from, for example, increasing the number of women in the dataset.^14^ The addition of fairness constraints in within a model can ensure that an AI model has fairness-aware training; this method also means that the model requires less upkeep through regular retraining.^17^ Both of these can be difficult to implement, either due to ongoing requirements or initial difficulty; however, when an AI has been showed to be biased, these can be easier than starting from scratch in many cases.

It is important to note that just because the training data may be biased, this does not necessarily imply that the model’s outputs will be biased. Often due to which relations the model learns as important, as not all relations will be learnt by the AI. This means that it is possible for AI to be used to highlight where current decisions may be occurring due to biased practices. For example, a potential fairness concern may be if a sepsis treatment model treated maternal sepsis patients differently, due to their different baselines. However, within this example the model did not identify the maternal patients any differently,^18^ although a clinician may well do so.

## Conclusion

We have introduced the concept of fairness in medical AI, discussed some causes of bias and discrimination in healthcare, and how these may affect AI models created now and in the future. We have highlighted a small number of potential solutions that can be implemented by either clinicians or AI specialists, and highlighted the two most implementable solutions, namely clinician awareness and AI identification of bias concerns.

There are a number of limitations of current fairness work in medical AI. Any fairness concerns need to be raised and highlighted before being checked, there are no methods that allow for generalised checks across different marginalised groups and scenarios. Intersectionality between marginalised groups, while possible, is largely theoretical and difficult to implement in practice at scale.

Fairness and medical AI has a lot of directions to still grow. Tt is important that, as models progress and are implemented, clinicians and patients are included throughout the process. This can help ensure, among other things, that fairness concerns are raised and caught early, before they have a negative impact on outcomes.

## CRediT authorship contribution statement

**Siân Carey:** Conceptualization, Methodology, Writing – original draft, Writing – review & editing. **Allan Pang:** Conceptualization, Methodology, Writing – original draft. **Marc de Kamps:** Conceptualization, Supervision, Writing – review & editing.

## Declaration of competing interest

The authors declare that they have no known competing financial interests or personal relationships that could have appeared to influence the work reported in this paper.
